# Supplementary figures and images for: The Relationship between Fat Mass Percentage and Glucose Metabolism in Children and Adolescents: A Systematic Review and Meta-Analysis
Source: Nutrients. 2022 May 28;14(11):2272. doi: 10.3390/nu14112272 (PMC9183098; doi:10.3390/nu14112272)

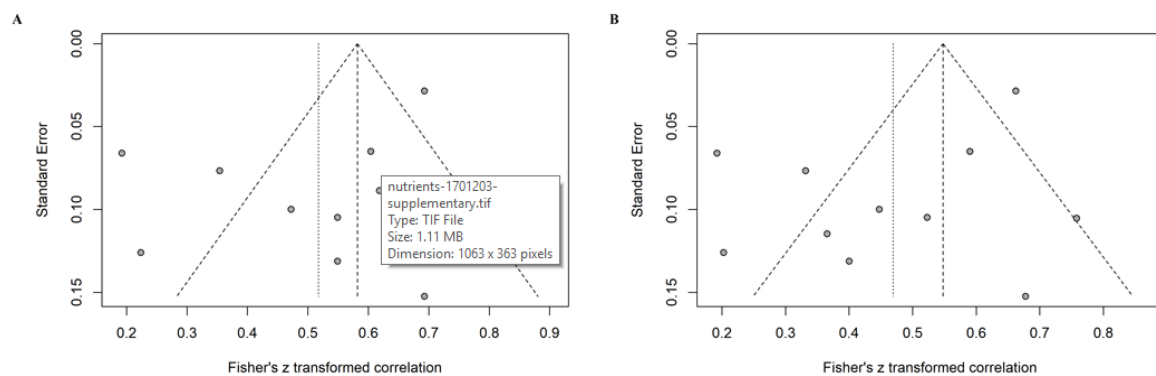

Figure S1. The funnel plots of the associations of FMP with INS (**A**) and HOMA-IR (**B**).

Supplement: Supplementary file 1 [file nutrients-14-02272-s001.zip › nutrients-1701203-supplementary.pdf]
